# Supplementary material for: MetaRibo-Seq measures translation in microbiomes
Source: Nat Commun. 2020 Jun 29;11:3268. doi: 10.1038/s41467-020-17081-z (PMC7324362; doi:10.1038/s41467-020-17081-z)
Supplement: Supplementary file 10 — Supplementary Data 7 [file 41467_2020_17081_MOESM10_ESM.zip › File2/Confidence_VeryHigh_Taxonomy/5525_out.krona.html]

Javascript must be enabled to view this page.

members
magnitude
magnitudeUnassigned
count
unassigned
taxon
rank

5525\_out

29

2
26
superkingdom

1224
1
phylum

subphylum
1
68525

class
29547
1

order
213849
1

family
1
72294

genus
194
1

species

SRS893227\_contig\_number\_contig-100\_11712.11713
204
1

1
1239
phylum

186801
1
class

186802
1
order

family
186803
1

genus
1164882
1

1
617123

SRS893227\_contig\_number\_contig-100\_8242.45075
species

24
203691
phylum

203692
24
class

24
136
order

24
137
family

genus
24
1

SRS019071\_contig\_number\_29698
157


SRS016969\_contig\_number\_contig-100\_828.211237SRS017209\_contig\_number\_19852SRS022143\_contig\_number\_36864SRS024441\_contig\_number\_29265SRS046496\_contig\_number\_9839SRS049389\_contig\_number\_contig-100\_5160.265434SRS057205\_contig\_number\_contig-100\_7670.42450SRS104384\_contig\_number\_14782SRS142787\_contig\_number\_contig-100\_722.224996SRS144644\_contig\_number\_contig-100\_2885.2886SRS893274\_contig\_number\_contig-100\_173.73571
species
11
1539298

species

SRS104087\_contig\_number\_21751
167
1


SRS062544\_contig\_number\_21358SRS147541\_contig\_number\_21125
species
2
69710

1
221027

SRS064014\_contig\_number\_contig-100\_308.154921
species


SRS015434\_contig\_number\_8970
species
1
58231

7
158

SRS019696\_contig\_number\_10518SRS075947\_contig\_number\_16589SRS077633\_contig\_number\_5227SRS143529\_contig\_number\_37785SRS144300\_contig\_number\_38633SRS144560\_contig\_number\_3613SRS144701\_contig\_number\_contig-100\_778.174327
species


SRS075870\_contig\_number\_contig-100\_34782.34783
1

2759
2
superkingdom

kingdom
4751
2

subkingdom
451864
2

phylum
2
4890

subphylum
2
147538

2
147548
class

order
2
5120

family
34371
2

genus
2
34372

2
34373

SRS050669\_contig\_number\_contig-100\_569.110645SRS144512\_contig\_number\_22587
species
